# New York State College

**Published:** 1896-11

**Authors:** 


### AMONG THE COLLEGES.

### NEW YORK STATE COLLEGE.

THE three classes will all have representations at the opening session of the New York State College, at Ithaca. The clinical advantages of this school have been a matter of concern to the profession in general, but it would seem that the clinical facilities of the school promise to attract a wide and varied clinic, to in every way provide for a thorough and efficient course.

